# Microstructure and Properties of ER50-6 Steel Fabricated by Wire Arc Additive Manufacturing

**DOI:** 10.1155/2021/7846116

**Published:** 2021-08-04

**Authors:** Qingxian Hu, Junyan Miao, Xiaoli Wang, Chengtao Li, Kewei Fang

**Affiliations:** ^1^School of Materials Science and Engineering, Jiangsu University of Science and Technology, Zhenjiang 212003, China; ^2^Suzhou Nuclear Power Research Institute, Suzhou 215004, China

## Abstract

In this paper, ER50-6 steel was fabricated by wire arc additive manufacturing (WAAM) with an A-W GTAW system. The microstructure, mechanical properties, and corrosion behaviors of ER50-6 steel by WAAM were studied. The results showed that, with the GMAW current increased, from the bottom to the top of the sample, the microstructure was fine ferrite and granular pearlite, ferrite equiaxed grains with fine grains at grain boundaries, and columnar ferrite, respectively. The average hardness in the vertical direction of samples 1# and 2# was 146 and 153 HV, respectively. The hardness of sample 2# increased because of the refinement of grain. The pores in the sample increased as the bypass current increased. The higher bypass current also has a deterioration effect on the corrosion behavior of ER50-6 steel.

## 1. Introduction

Wire arc additive manufacturing (WAAM) becomes the core of the new industrial era. It has many advantages such as high deposition rate [1], low equipment cost, high material utilization, and environmental friendliness. It is a versatile and cost-effective method to fabricate complex parts and large-sized components [2]. It included three types of processes, namely, gas metal arc welding (GMAW), gas tungsten arc welding (GTAW), and plasma welding (PAW). In these processes, the arc is used as the heat source, and the wire is used as additive manufacturing materials. Compared to the process powder-based techniques, it has higher efficiency and lower production cost. As is known, there have been many research reports about WAAM, such as steel [3–5], Ti alloys [6], and steel and Al alloys [7, 8]. In the additive manufacturing, some defects, such as uncontrolled grain size, tensile residual stress, cracks, and delaminations [9], are very easy to produce. In order to widen the adoption of WAAM in diverse industries, some scientists studied how to eliminate the defects. Colegrove et al. [10] combined high-pressure rolling with WAAM and reported that peak residual stress was reduced and refined microstructure was obtained. Martina et al. [11] evaluated fatigue crack propagation behavior in WAAM Ti-6Al-4V using the numerical simulation method. In fact, during the process of WAAM, the main reason for various defects is due to excessive heat input. Therefore, how to maintain a high deposition rate, avoid excessive heat input, and maintain arc stability has become an urgent problem in the process of wire arc additive manufacturing. In order to reduce heat input, researchers used different processes for additive manufacturing. Li et al. [12] used cold metal transfer (CMT) to fabricate a higher and thinner layer by preheating the wire to reduce heat input. Based on compulsively constricted WAAM (CC-WAAM) proposed by Liu et al. [13], Rodrigues et al. [14] proposed a new process named ultracold-wire and arc additive manufacturing (UC-WAAM) that can reduce the process temperature, and it can create a hollow part without any support structure. However, it was not easy to control droplet transition during these processes.

**Figure 1:**
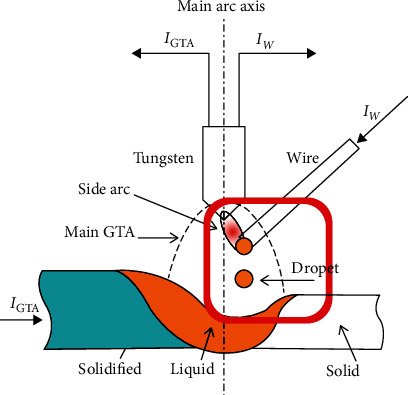
The schematic diagram of the A-W GTAW process [15].

Obviously, in addition to reducing heat input, the heat input should be controlled independently. As is known, gas metal arc welding (GMAW) has a high deposition rate. Gas tungsten arc welding (GTAW) has low heat input, and it is stable and easy to operate. The advantages of GMAW and GTAW should be fully utilized. Zhang and his colleagues [15] have proposed an innovative arcing-wire gas tungsten welding (A-W GTAW). The schematic diagram of the A-W GTAW process is shown in Figure 1. In the A-W GTAW system, between the tungsten and the feeding wire, a side arc was inside the main GTAW. In their study paper, the process was described in detail to reduce heat input to the substrate. The feeding wire can be directly and quickly melted at high speeds. Compared with the traditional GMAW and GTAW, the A-W GTAW process makes full use of the advantages of GMAW and GTAW and achieves a high deposition rate, and it has a stable arc to be operated [15]. Therefore, it is very meaningful to attempt this process for additive manufacturing. ER50-6 wire is widely used for welding ordinary carbon steel, carbon structural steel for automobile manufacturing, and low alloy and high strength structural steel for hull and pressure vessel. This work attempted to use this process to fabricate ER50-6 steel thin wall, and the microstructure, mechanical properties, and corrosion resistance of the prepared parts were analyzed. This study will provide the research foundation for the wire arc additive manufacturing of the large components.

## 2. Experimental Details

The substrate was Q235. ER50-6 welding wire was used to additively manufacture samples.  Table 1 shows the chemical composition of Q235 steel and ER50-6 welding wire. In order to remove the rust and oil on the surface of Q235, an angle grinder and acetone were used.

**Figure 2:**
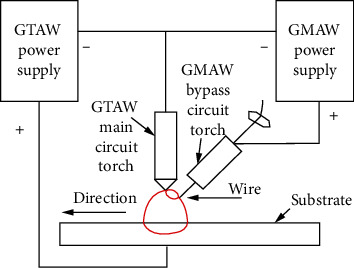
The circuit diagram of the A-W GTAW additive manufacturing system.

Based on Liu et al.'s study [13], Figure 2 shows the circuit diagram of the A-W GTAW additive manufacturing system. A special WSM-315C argon arc welding machine (Aotai, Shandong, China) and general GMAW-500P gas protection welding machine (Aotai, Shandong, China) are used to build the system of A-W GTAW. The tungsten electrode is connected to the negative power of the GTAW and GMAW welding machines. The positive electrode of GTAW and GMAW is connected to the substrate and the feeding wire, respectively.

In the process of additive manufacturing, with the increase of surfacing layers, the weld surplus height increased gradually. In order to control the consistency of welding gun height from the substrate, a high-precision lifting platform was used to adjust the height, and the adjustment range was 0-60 mm.

**Figure 3:**
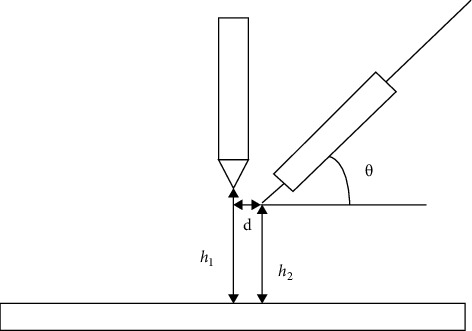
The position of the welding torch.

As shown in Figure 3, the distance marked *h*_1_ between the substrate and the tungsten electrode tip was 9 mm. The feeding wire located directly below the tungsten electrode was 7.5 mm away from the additive manufacture layers. The horizontal distance marked *d* between the tungsten electrode and the feeding wire was 2 mm, and the angle between the GTAW electrode and the horizontal electrode was 35°. The protective gas was 99.99% pure argon. Single-pass multilayer surfacing welding was carried out on the Q234 substrate, and the time interval of each layer was 10 minutes. The deposition direction of each layer was the same. Based on many experiments, two good samples (prepared by two different processes) were selected for comparative analysis of microstructure and properties.  Table 2 shows the process parameters of additive manufacturing of ER50-6.

**Figure 4:**
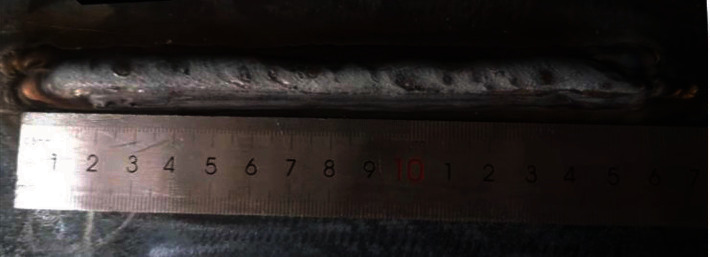
The macro morphology of additively manufactured ER50-6.

Figure 4 shows the macro morphology of additively manufactured ER50-6. The cross-sectional samples were obtained by wire cutting of the additively manufactured ER50-6, and samples for microstructure observation and performance test were prepared. First, the three samples were grinded with 200#, 600#, and 1000# sandpaper and polished with 2.5-mesh polishing paste. Samples prepared for microstructure observation were corroded with 4% nitric acid alcohol.

The microstructures of the two samples were observed using a Nikon Epiphot300 optical microscope (OM) (Nikon, Japan) and a scanning electron microscope with field emission (FESEM, SU-70, Hitachi, Japan). DHV-1000 model of hardness tester (Shanghai Shangcai Testermachine Co., LTD., China) was used to test the Vickers hardness of samples. The test load was 10 N, and the last time was 10 s. CSM NHT2 Nanoindenter (Anton Paar) was used to measure the elastic properties of samples 1# and 2# with 10 mN of the maximum load and 20 mN·min^−1^ of the loading/unloading rate. The depth recovery ratio (*ŋ*_h_) was used to characterize the elastic behavior of samples. The value of *ŋ*_h_ was defined as follows [16, 17]:

(1) $$\begin{array}{c} ŋ=\frac{h_{\max}-h_{\text{r}}}{h_{\max}}, \end{array}$$

in which *h*_max_ was the maximum depth of penetration and *h*_r_ was the residual depth after unloading. The elastic modulus (EIT) of samples can be obtained directly by the test. The elastic modulus value was greater, which meant that the material deformation was not easy to happen, that is, it was more brittle.

The tribocorrosion tests were performed in 3.5% NaCl solution at 25°C using an MSR-2T tribometer (Lanzhou Institute of Chemical Physics, Chinese Academy of Sciences, Lanzhou, China). In the present work, 25 × 15 mm^2^ surface area of tested samples was in contact with the electrolyte throughout the tribocorrosion testing. The tribocorrosion test was conducted at a speed of 1 mm/s with a normal load of 20 N. The electrochemical behaviors of the tested specimen were conducted using a Reference 600+ electrochemical workstation (Gamry Instruments, Inc. USA) and a three-electrode electrochemical cell, with a saturated calomel electrode (SCE) as the reference electrode. Potentiodynamic polarization was measured after friction test was performed for 10 min and scanned at a constant rate of 1 mV/s from −400 mV below the corrosion potential and terminated when a current value of 10 mA was reached.

## 3. Results and Discussion

### 3.1. Macroscopic Morphology Observation

**Figure 5:**
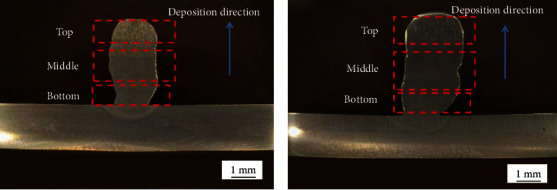
OM of the additively manufactured samples (a) 1# and (b) 2#.

The morphology of the tested samples is shown in Figure 5. As is seen from Figure 5, the depth and height of the melting pool increased with the increase of GMAW current, indicating that the amount of the melted substrate increased [18]. It indicated that the deposition of arc additive was effectively improved by increasing GMAW current. On the other hand, the substrate deformation was more serious with the increase of GMAW current. In the following work, metallographic analysis and SEM observation experiments were carried out on samples 1# and 2#.

According to the deposition direction, samples were divided into three regions, top part, middle section, and bottom part, next to the fusion zone, as shown in Figure 5. The three regions were analyzed by optical microscopy and SEM in the paragraphs below.

### 3.2. Optical Microscopic (OM) Analysis

**Figure 6:**
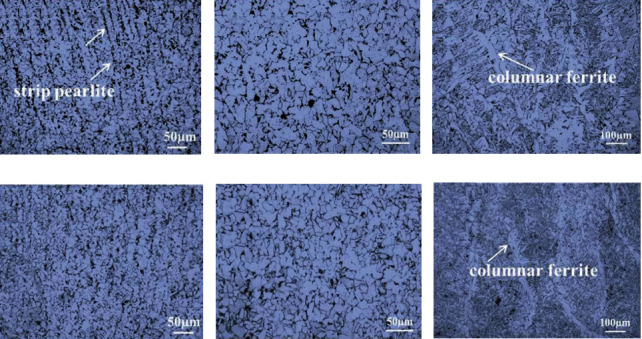
OM of the different positions of samples 1# and 2#. The images of the (a, d) bottom, (b, e) middle, and (c, f) top sections of samples 1# and 2#, respectively.

The microstructure transformation of ER50-6 steel can be divided into three types: ferrite phase transformation, pearlite phase transformation, and bainite phase transformation.  Figure 6 shows the OM images of the different positions of samples 1# and 2#. It was seen that there was more strip pearlite in Figure 6(a) than in Figure 6(d). Because bypass current *I*_2_ of sample 2# was larger than that of sample 1#, more heat input homogenizes the ingredients so that strip pearlite was not as obvious in Figure 6(d). The matrix of Figure 6(d) consisted of fine ferrite and granular pearlite. From Figures 6(b) and 6(e) in the middle of samples 1# and 2#, respectively, it can be seen that ferrite grains were equiaxed, especially on the grain boundary, and the grains in Figure 6(e) were smaller than those in Figure 6(b). During additive manufacturing, continuous thermal cycling resulted in recrystallization of grains. These grains can play a role in fine-grain strengthening. On the top of sample 1#, from Figure 6(c), it can be seen that there was columnar ferrite located at the grain boundary and acicular ferrite and pearlite located in grains. On the top of sample 2#, from Figure 6(f), it indicated that there was narrower columnar ferrite than that of sample 1#. It was that the different microstructures of the two samples would be leading to different mechanical properties, which was the result of rapid solidification [19].

### 3.3. SEM Analysis

**Figure 7:**
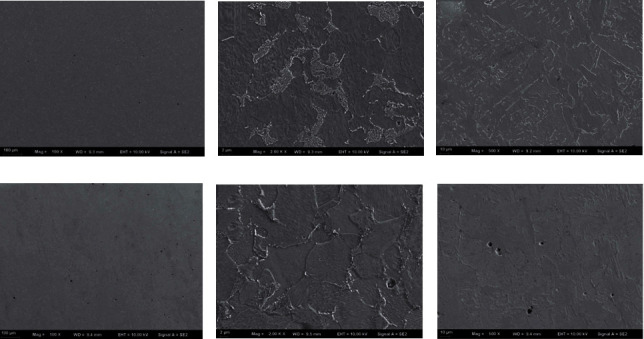
The SEM images of (a–c) sample 1# and (d–f) sample 2#. The images of the (a, d) bottom, (b, e) middle, and (c, f) top sections of samples 1# and 2#, respectively.

The SEM images of samples 1# and 2# are shown in Figure 7. Figures 7(a) and 7(d), 7(b) and 7(e), and 7(c) and 7(f) are the images of the bottom, middle, and top section of samples 1# and 2#, respectively. As seen from Figures 7(a) and 7(d), there were regular circular pores. The defects of pores are often found in components fabricated by additive manufacturing [20]. It was mainly due to the evaporation of metal elements in the additive manufacturing process and the instability of the molten pool caused by the increase of bypass current *I*_2_. The number of pores in sample 2# was significantly higher than that in sample 1#. From Figure 7(b), the lamellar structure of pearlite can be seen. However, in Figure 7(e), this structure cannot be seen because the pearlite became smaller than that in sample 1#. At the top of sample 2#, from Figures 7(c) and 7(f), it can be seen that the pores were bigger than those on the top of sample 1#. The increase in the number and volumes of these pores was mainly due to the increase of current density.

### 3.4. Mechanical Test

#### 3.4.1. Hardness Test

**Figure 8:**
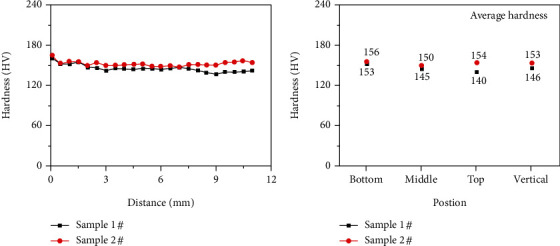
Hardness curves of samples 1# and 2#. (The horizontal distance was from the center axis of the fusion line to the top of samples.).

Figure 8 presents the microhardness (HV) distribution of samples 1# and 2# in the cross section, as shown in Figure 5.  Figure 8(a) shows the hardness curve of samples 1# and 2# from the center axis of the bottom to the top. The vertical direction is consistent with the deposition direction. The hardness of the two samples was similar to each other at the bottom and in the middle, while hardness curves at the top of samples varied.  Figure 8(b) shows the average hardness in different areas of samples. The average hardness of sample 1# and sample 2#, at the bottom, in the middle, and at the top, was 153 and 156 HV, 145 and 150 HV, and 140 HV and 154 HV, respectively. It can be seen that the average hardness values of the bottom and middle of the two samples had little difference. Their difference was mainly reflected in the average hardness at the top of samples. The average hardness in the vertical direction of samples 1# and 2# was 146 and 153 HV, respectively. The hardness of sample 2# was higher than that of sample 1#. Combined with the analysis results in Figure 6, it can be concluded that the lowest hardness at the top of sample 1# was due to the presence of large columnar ferrite. Grain refinement in the middle and at the top of sample 2# was the reason why the hardness of sample 2# was higher than that of sample 1#. The analysis results were consistent with those in Figure 6.

#### 3.4.2. Nanoindentation Characterization

**Figure 9:**
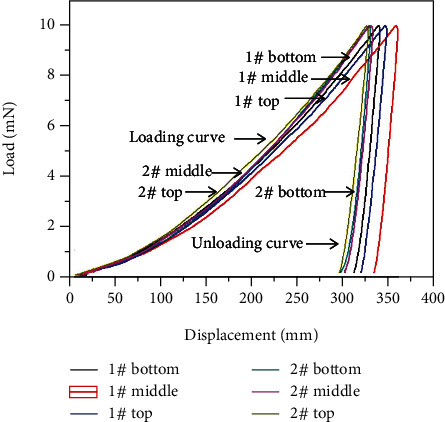
The load-displacement curves (*P*-*h*) of different parts for samples 1# and 2#.

The nanoindentation curves of different parts for samples 1# and 2# are shown in Figure 9. The maximum depth (*h*_max_) of indentation with 10 mN load of the middle of samples 1# and 2# was 358.86 nm and 331.16 nm, respectively. The values of the parameters, including the maximum depth (*h*_max_), residual depth (*h*_r_), depth recovery ratio (*ŋ*_h_), and elastic modulus (EIT) extracted from Figure 9, are presented in Table 3. *h*_max_ for sample 1# at the bottom, middle, and top was higher than that for sample 2#. It indicated that sample 1# had a higher resistance to plastic deformation than sample 2#.

**Figure 10:**
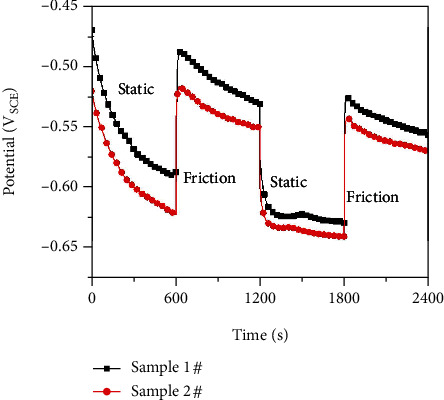
*E*_corr_ vs. time for the tested samples in 3.5% NaCl solution under static and friction conditions.

The corrosion potentials (*E*_corr_) of the tested samples measured under static and friction conditions in 3.5% NaCl solution are shown in Figure 10. During the measurements, the static and friction conditions were periodically switched. In the first static period of 30 min, *E*_corr_ of the two samples decreased continuously. When the friction started, *E*_corr_ sharply shifted to a more positive potential, followed by the slow shifting to a negative direction. Once the friction stopped, *E*_corr_ suddenly dropped. At the second static immersion period and friction period, the response of *E*_corr_ was identical to that of the first test cycle. Whether in static or friction conditions, *E*_corr_ of sample 1# was always higher than that of sample 2#. The stirring effect of friction can accelerate the diffusion of oxygen and lead to the increase of their active dissolution [18, 21–23].

**Figure 11:**
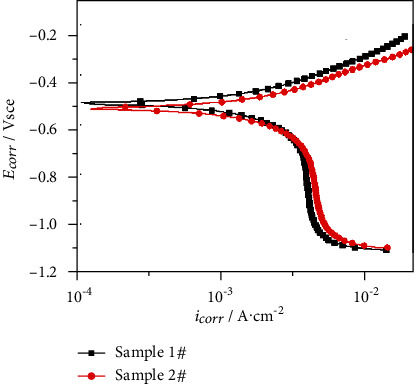
Potentiodynamic polarization curves for the two tested samples in 3.5% NaCl solution under friction condition.

Figure 11 shows the polarization curves for the two tested samples in 3.5% NaCl solution under friction conditions. As seen from Figure 11, both alloys exhibited the active corrosion behavior. The electrochemical response of the two samples is similar. The corrosion potential (*E*_corr_) and corrosion current density (*i*_corr_) obtained from Figure 11 are listed in Table 4. As is seen in Table 4, sample 2# exhibited a relatively higher corrosion current density. This suggested that a higher bypass current accelerated the corrosion rate of the specimens in 3.5% NaCl solution. One reason was that sample 2# had more defects than sample 1#, and the other was that the refinement of sample 2# provided more channels for corrosion [24–28].

## 4. Conclusions

The conclusions can be drawn as follows:

1. ER50-6 steel was fabricated by wire + arc additive manufacturing based on the A-W GTAW system. The deposition rate of arc additive can be significantly improved by increasing bypass current through cross-sectional analysis of additive samples by an optical microscope
2. The microstructure of the two samples in the middle was ferrite grains which were equiaxed. With the GMAW current increased, from the bottom to the top of the sample, the microstructure was fine ferrite and granular pearlite, ferrite equiaxed grains with fine grains at grain boundaries, and columnar ferrite, respectively. The average hardness in the vertical direction of samples 1# and 2# was 146 and 153 HV, respectively. The hardness of the sample increased because of the refinement of grains
3. The higher bypass current had a deterioration effect on the corrosion behavior of ER50-6 steel because it can produce more defects and make grain refinement that provided more channels for corrosion

## Figures and Tables

**Table 1:** Chemical composition of Q235 steel and ER50-6 wire (wt.%).

Elements	Mn	Si	S	P	C	Cu	Fe
Q235	0.30	0.15	0.035	0.015	0.17	/	Bal.
ER50-6	1.40-1.85	0.80-1.15	≤0.035	≤0.035	0.06-0.15	≤0.50	Bal.

**Table 2:** Main technological parameters of additive manufacturing.

The process parameters	Sample 1#	Sample 2#
Main current *I*_1_ (A)	150	150
Bypass current *I*_2_ (A)	150	200
Tungsten electrode diameter (mm)	3	3
Angle of tungsten electrode *θ* (°)	50	50
Gas flow of GTAW (L·min^−1^)	15	15
Gas flow of GMAW (L·min^−1^)	12	12
Welding speed (mm·min^−1^)	160	160
Layer numbers	12	12

**Table 3:** The parameters of indentation from the load-displacement curves in Figure 8.

Sample	Location	*h*_max_ (nm)	*h*_r_ (nm)	*ŋ*_h_	EIT (GPa)
1#	Bottom	339.02	316.07	0.08	258.22
	Middle	358.66	338.82	0.06	286.09
	Top	346.44	323.59	0.07	252.16
2#	Bottom	328.77	308.83	0.06	316.44
	Middle	331.16	311.21	0.06	312.9
	Top	326.26	302.83	0.07	262.61

**Table 4:** Electrochemical parameters extracted from potentiodynamic polarization curves (Figure 11).

	*E*_corr_ (mV_SCE_)	*i*_corr_ (A·cm^−2^)
Sample 1#	−543 ± 5	1.13 ± 0.04 × 10^−3^
Sample 2#	−514 ± 6	1.32 ± 0.07 × 10^−3^

## Data Availability

The data used to support the findings of this study are available from the corresponding author upon request.
